# Establishment of the prognostic index of lung squamous cell carcinoma based on immunogenomic landscape analysis

**DOI:** 10.1186/s12935-020-01429-y

**Published:** 2020-07-20

**Authors:** Jianguo Zhang, Jianzhong Zhang, Cheng Yuan, Yuan Luo, Yangyi Li, Panpan Dai, Wenjie Sun, Nannan Zhang, Jiangbo Ren, Junhong Zhang, Yan Gong, Conghua Xie

**Affiliations:** 1 Department of Radiation and Medical Oncology, Zhongnan Hospital of Wuhan University, Wuhan, 430071 Hubei China; 2 Department of Biological Repositories, Zhongnan Hospital of Wuhan University, Wuhan, 430071 Hubei China; 3 Hubei Key Laboratory of Tumour Biological Behaviors, Zhongnan Hospital of Wuhan University, Wuhan, 430071 Hubei China; 4 Hubei Cancer Clinical Study Center, Zhongnan Hospital of Wuhan University, Wuhan, 430071 Hubei China; 5 Human Genetics Resource Preservation Center of Hubei Province, Human Genetics Resource Preservation Center of Wuhan University, Zhongnan Hospital of Wuhan University, Wuhan, 430071 Hubei China; 6 Department of Occupational and Environmental Health, School of Public Health, Qingdao University, Dengzhou Road 38, Qingdao, 266021 China

**Keywords:** Lung squamous cell carcinoma, Immunogenomic landscape, Prognostic index, Bioinformatics

## Abstract

**Background:**

The incidence of lung squamous cell carcinoma (LUSC) increased substantially in recent years. Systematical investigation of the immunogenomic pattern is critical to improve the prognosis of LUSC.

**Methods:**

Based on the TCGA and GEO dataset, we integrated the immune-related genes (IRGs) expression profile and the overall survival (OS) of 502 patients with LUSC. The survival-related and differentially-expressed IRGs in LUSC patients were evaluated by univariate cox regression and LASSO regression analysis. By applying multivariate cox analysis, a new prognostic indicator based on IRGs was established. We also used CIBERSORT algorithms and TIMER database to analyze immune infiltration of LUSC. Both gene set enrichment analysis (GSEA) and principal component analysis (PCA) was carried out for functional annotation. With the assist of computational biology, we also investigated the latent properties and molecular mechanisms of these LUSC-specific IRGs. We analyzed the correlation between immune checkpoints and risk score.

**Results:**

A novel prognostic model was established based on 11 IRGS, including CXCL5, MMP12, PLAU, ELN, JUN, RNASE7, JAG1, SPP1, AGTR2, FGFR4, and TNFRSF18. This model performed well in the prognostic forecast, and was also related to the infiltration of immune cells. Besides, the high-risk groups and the low-risk groups exhibited distinct layout modes in PCA analysis, and GSEA results showed that different immune status among these groups.

**Conclusions:**

In summary, our researches screened out clinically significant IRGs and proved the significance of IRG-based, individualized immune-related biomarkers in monitoring, prognosis, and discern of LUSC.

## Background

Lung cancer is the principal reason for tumor-related deaths, with 1.7 million deaths worldwide annually [1]. Non-small cell lung cancer (NSCLC) approximately take up 85% of all lung cancer cases [2]. LUSC is one of the major subtypes of NSCLC, accounting for approximately 25% to 30% of NSCLC [3]. LUSC is usually located in the hilum of lung and usually occurs in the proximal bronchus, and it is more likely to invade larger blood vessels [4–6]. Although the technologies in early detection, targeted therapy, and chemotherapy were substantially improved during the last decades, the OS of LUSC patients remains poor [7].

Cancer immunotherapy has been the main driving force of personalized medicine, by activating the immune system oppose cancer [8, 9]. In recent decades, immunotherapy was included in the treatment guidelines for multiple cancers [10, 11]. T cell is an important component of tumor immunity [12]. The standard treatment of immunotherapy is to promote T cell functionality in tumors [13], and the studies on immunotherapy focus on the recruitment of cancer-infiltrating T cells [14]. In lung cancer, cancer-infiltrating CD4 + T cells have a vital impact on the immune response [15]. CD4 + T cells were reported to recruit CD8 + T cells to the tumor site [16] and infect mucosa [17]. In addition, they were necessary to inhibit angiogenesis at the tumor sites [18, 19]. Recently, several immune checkpoint inhibitors were found to enhance cytotoxic competence by targeting PD-1 ligand 1 (PD-L1), cytotoxic T lymphocyte antigen-4 (CTLA-4), and programmed cell death protein 1 (PD-1). They also had significant clinical effects on LUSC [20]. PD-1 antibodies, Nivolumab and Pembrolizumab, as well as PD-L1 antibody Atezolizumab, were allowed for NSCLC therapy [21, 22]. With the development of immune therapy, the relationship between immune cell and tumor has become a hot topic [23, 24]. The prognostic value of IRGs was comprehensively explored to utilize personalized immune signals for optimal prognostic evaluations in non-squamous NSCLC patients [25]. However, the prognostic significance and clinical correlation of IRGs in LUSC remain to be explored.

We combined clinical information with IRG expression profiles to evaluate the OS of LUSC patients. The prognostic landscape and expression status of IRGs were systematically analyzed, and individual prognostic characteristics for patients with LUSC were developed. We found that 11 IRGs were significantly correlated with prognosis, and established a new independent prognostic model based on these genes. This model also well predicted immune cell infiltration in LUSC. Our study provided a potential model and biomarkers for further immune-related work and personalized medicine for the treatment of LUSC.

## Materials and methods

### Data collection and processing

The RNA-seq FPKM data of LUSC, containing corresponding clinical data, were downloaded from the TCGA [26], which included 502 LUSC tissues and 49 normal tissues. The dataset (GSE73403) on LUSC with survival data was downloaded from the GEO database as a validation set. This dataset contained 69 tumor samples. IRG list in the ImmPort database has been exported [27]. These genes have been identified as active participants in immune processes. We then screened the immune genes shared by TCGA and GEO datasets. The differentially expressed genes (DEGs) in LUSC and its adjacent normal tissues were analyzed by the R software limma package. The log2 | fold change | > 1 and false discovery rate (FDR) < 0.05 were set as the cut-off value.

### Gene ontology and KEGG pathway analysis

To verify whether the DEGs were related to immune, GO and KEGG enrichment analysis were used. First, the org.Hs.e.g.db package was used to convert the gene symbol into entrezID. Then, GO and KEGG enrichment analysis were performed using the clusterProfiler package. P < 0.05 was considered as statistical significance. Finally, the GOplot package was used to draw the bar chart of GO and circle diagram of KEGG.

### Univariate COX analysis and LASSO analysis

To get survival- and immune-related genes, we integrated the expression of IRGs with the OS of LUSC patients. IRGs were then analyzed by univariate COX regression analysis with continuous variables (P < 0.05). These survival- and immune-related genes were integrated into least absolute shrinkage and selection operator (LASSO) regression, which was calculated by the glmnet package of R software with 1000 runs [28].

### Survival analysis

The patients whose follow-up time was less than 30 days were removed from the survival analysis. 470 patients were analyzed. Multivariate survival analysis was performed to inspect the overall effect of the IRGs on prognosis using the R software survival package. Finally, the prognostic model of LUSC was established based on the multivariate co-efficiency multiplied by expression data. The formula was as follows:

$$\begin{aligned} {\text{Risk score}} & \, = \,\alpha {\text{gene}}\left( {\text{a}} \right)\, \times \,{\text{gene expression}}\left( {\text{a}} \right)\, + \,\alpha {\text{gene}}\left( {\text{b}} \right) \\ & \,\, \times \,{\text{gene expression}}\left( {\text{b}} \right)\, + \, \cdots \; + \alpha {\text{gene}}\left( {\text{n}} \right)\, \times \,{\text{gene expression}}\left( {\text{n}} \right). \\ \end{aligned}$$

The survminer package of R software was used to apply the Kaplan–Meier curve to investigate the connection amid IRGs and prognosis. Univariate analysis and multivariate analysis were used to explore independent prognostic factors of LUSC patients. Survival ROC R Software package was used to calculate the area under the curve (AUC) to verify the manifestation of prognostic characteristics [29]. In addition, we drew a nomogram including the clinical factors and risk scores. The calibration curve and ROC curve were painted to illustrate the accurateness of this model in predicting the survival of LUSC patients.

### Validation of the immune-related genes

To investigate the expression of IRGs in distinct cancers, the Oncomine database was utilized to analyze the expression levels of the hub gene in tumor tissues and normal tissues. The Human Protein Atlas database was used to verify the protein function of IRGs by immunohistochemistry. The correlations between IRGs and clinical factors were also analyzed.

### Molecular characteristics of immune-related genes

We recognized the mutations in IRGs through Cbioportal database [30–32]. Cistrome cancer database is a resource for predicting transcription factor (TF) targets and enhancers in cancer [33]. We downloaded tumor-related TFs from this database and acquired differential expression TFs (log2 | fold change | > 1 and FDR < 0. 05). Through correlation analysis (corFilter > 0.4 and P < 0.001), the association between IRGs and TFs were established. Then we utilized Cytoscape to visualize this relationship.

### Analysis of the difference between high-risk and low-risk patients

We built the immune-related gene-based prognostic index (IRGPI) on the basis of the multivariate cox regression coefficient multiplied by expression data. According to the median PI value, the patients were classified into the high-risk group and low-risk group. Principal component analysis (PCA) was utilized to analyze the grouped samples and expression patterns and gene set enrichment analysis (GSEA) was carried out to evaluate different functional phenotypes between low-risk and high-risk groups [34].

### Analysis of the relationship between immune cell infiltration and immune-related genes

Based on all the genes of two cohorts, we used the CIBERSORT software package to evaluate the proportion of 22 leukocyte subtypes. The perm was set to 1000. The samples with P < 0. 05 in the results of CIBERSORT analysis were delivered for further investigate. Mann–Whitney U test was performed to contrast the difference of similar leukocyte subtypes between the low-risk group and the high-risk group. In addition, we also used TIMER database to calculate the relationship between IRGs and immune cell infiltration. TIMER reanalyzed gene expression data to assess the infiltrating levels of 6 immune cell subtypes, including CD4 + T cells, B cells, CD8 + T cells, neutrophils, macrophages, and dendritic cells. Therefore, it could be utilized to validation the connections between hub IRGs and immune cell infiltration. To explore the relationship between IRGs and immune checkpoints, we calculated the correlation between IRGs and 30 immune checkpoint genes.

## Results

### Identification of differentially expressed IRGs

**Fig. 1:**
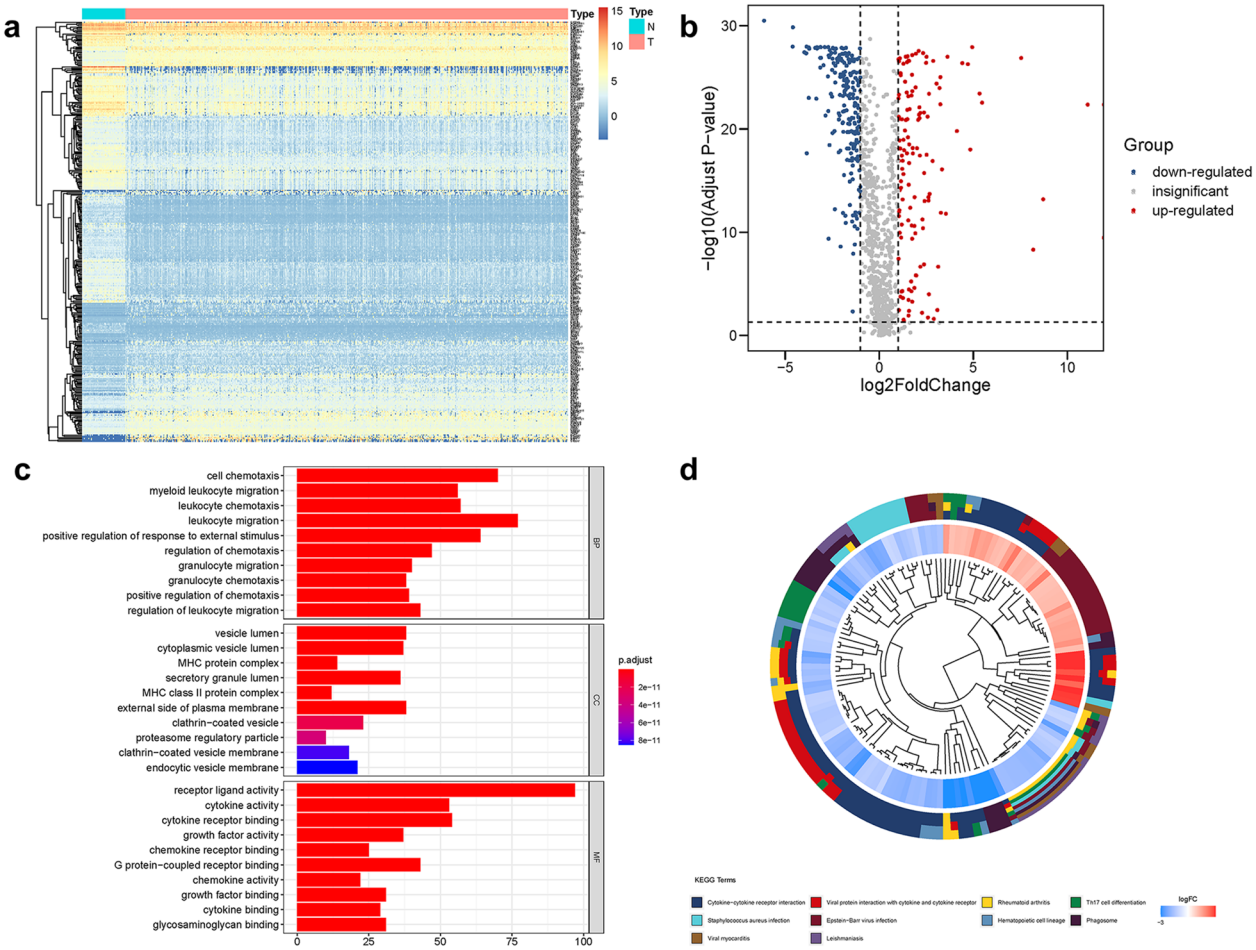
Differentially expressed and functional enrichment analysis. **a** Heatmap of differentially expressed IRGs. **b** Volcano plot of differentially expressed IRGs. **c** GO analysis. **d** KEGG analysis

**Fig. 2:**
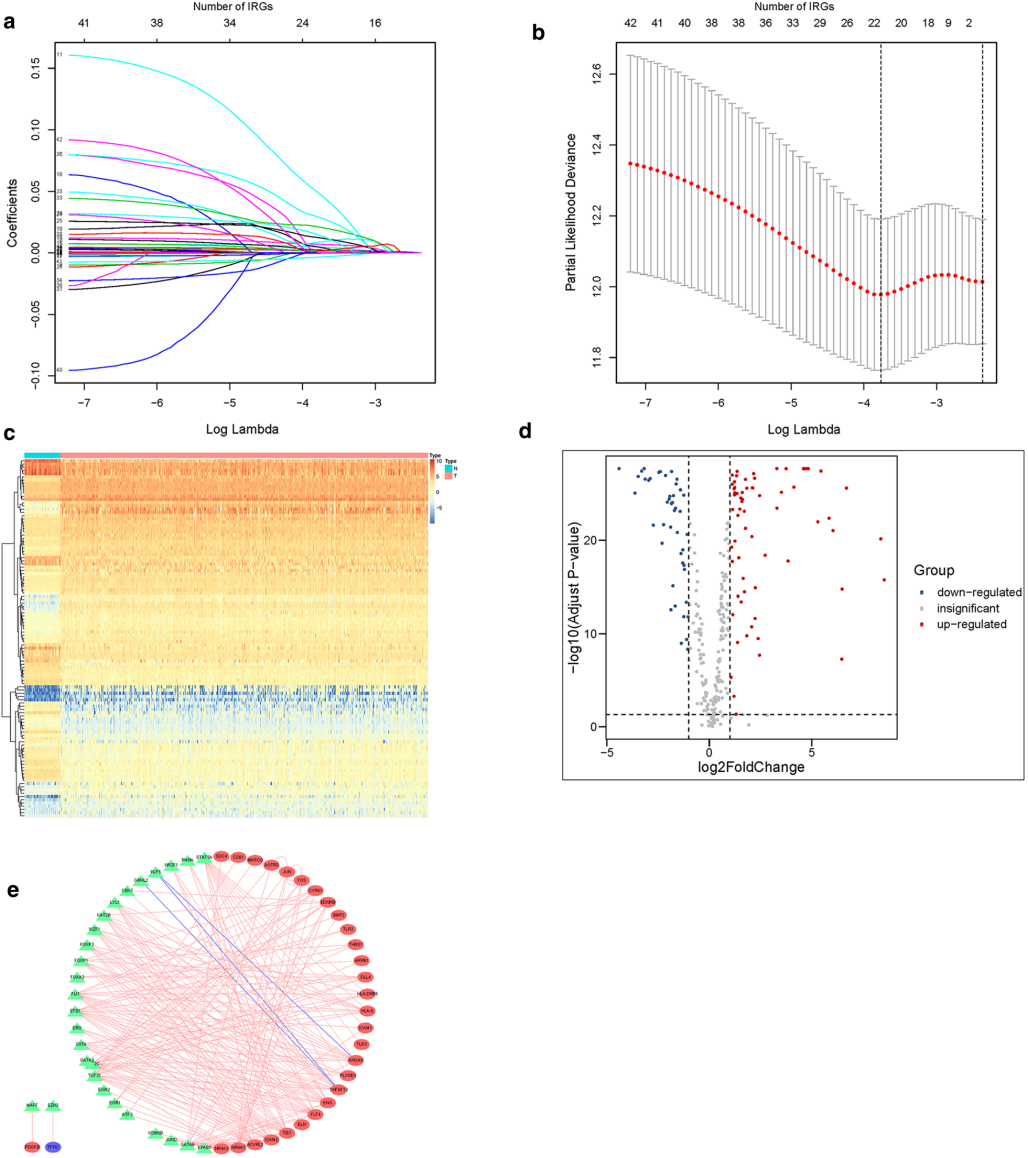
Identification of core prognosis concerning IRGs and TF network. **a** LASSO coefficient. **b** A graph of the error rate of cross-validation. **c** Heatmap of differentially expressed TFs. **d** Volcano plot of differentially expressed TFs. **e** Regulatory network built on the basis of IRGs and TFs. The red, green, and purple circles represent up-regulated IRGs, TFs, and down-regulated IRGs

According to the list of IRGs from the ImmPort database, 355 differentially expressed IRGs were identified, containing 135 upregulated and 220 downregulated (Fig. 1a, b). The results of GO analysis and KEGG analysis confirmed that the differential genes were related to immune (Fig. 1c, d). Through univariate COX regression analysis, 42 differentially expressed IRGs (P < 0.05) were notably associated with clinical outcomes. Then we used LASSO regression analysis to select these survival-related IRGs. As illustrated in Fig. 2a, b, Twenty-one IRGs were involved in the classifier.

### Transcriptional factor regulatory network

To investigate the latent regulatory mechanism of these IRGs expressions, we obtained differentially expressed TFs between LUSC and normal tissues using data downloaded from the Cistrome database. 111 differentially expressed TFs were identified (Fig. 2c, d). We built an interaction network on the basis of these 111 TFs and the 42 IRGs. The regulatory map showed the relationships between these IRGs and TFs (Fig. 2e).

### Evaluation of clinical outcome*s*

**Fig. 3:**
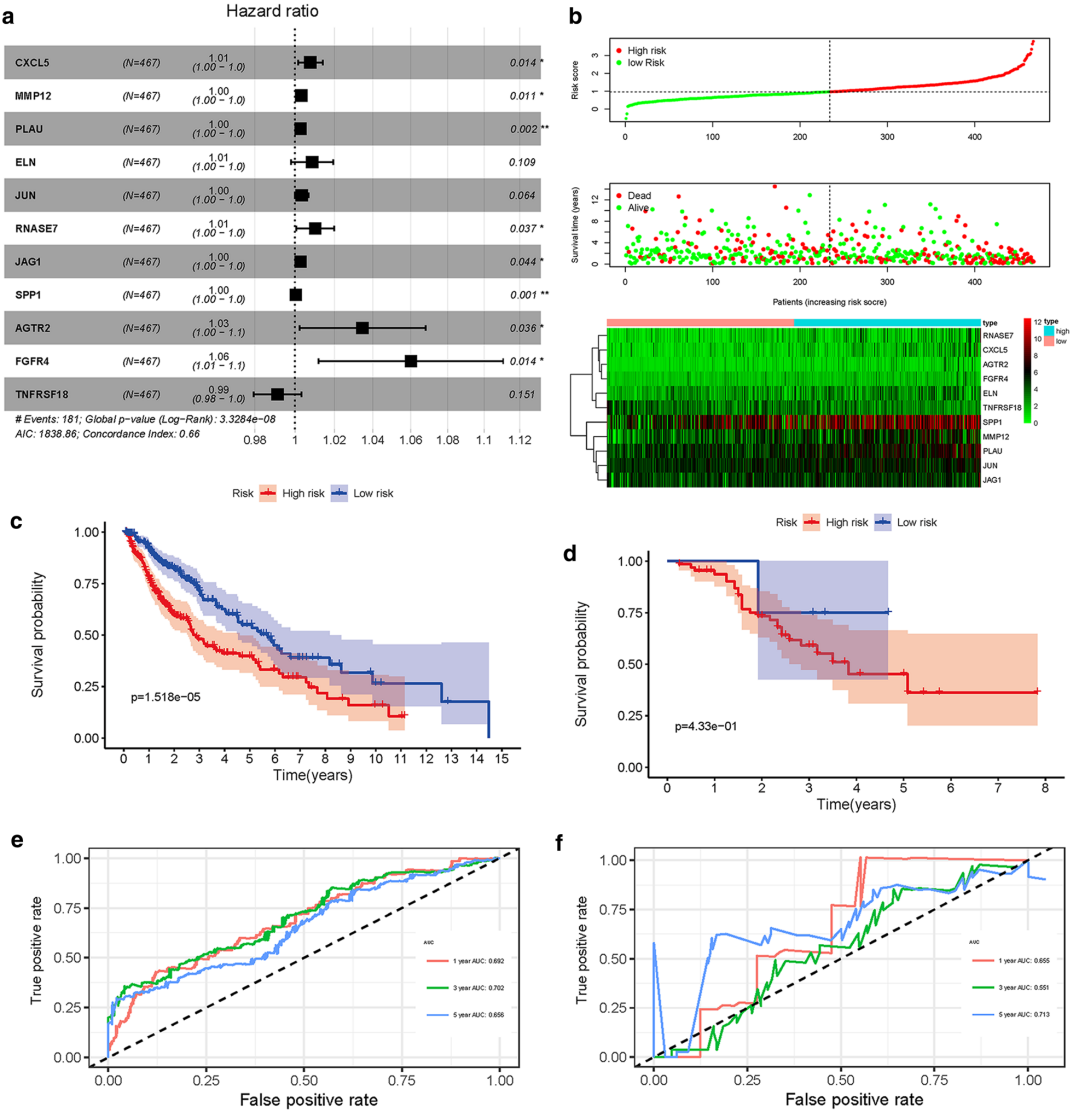
Overall survival of the low- and high-risk groups. **a** Forest plot of hazard ratios exhibiting the prognostic worth of IRGPI. **b** Survival conditions of LUSC patients. **c** K-M survival curve of TCGA cohort. **d** K-M survival curve of GEO cohort. **e** ROC curve verifies the accuracy of the model in predicting the 1-, 3-,5-year survival rates of LUSC patients in the training set. **f** ROC curve verifies the accuracy of the model in predicting the 1-, 3-, 5-year survival rates of LUSC patients in the validation set

On the basis of IRGs, we built a prognostic model through multivariate cox regression analysis. As shown in Fig. 3a, the hazard ratio of most genes was greater than 1, indicated that the high expression level of these genes implied poor prognosis. With the increased in the risk score, the number of deaths was also increased (Fig. 3b). The calculation formula of the risk score was shown as follows:

$$\begin{aligned} {\text{Risk score}}\, & = \,\left[ {{\text{CXCL5}}\,*\,\left( {0.00 7 7 2 3 9 1 6} \right)} \right]\, + \,\left[ {{\text{PLAU}}\,*\,\left( {0.00 2 7 7 3 1 9 6} \right)} \right]\, + \,\left[ {{\text{RNASE7}}\,*\,\left( {0.0 10 1 5 1 8 4 6} \right)} \right] \\ & \, + \,\left[ {{\text{MMP12}}\,*\,\left( {0.00 3 1 2 5 9 3} \right)} \right]\, + \,\left[ {{\text{ELN}}\,*\,\left( {0.00 8 6 7 8 3 8 1} \right)\, + \,} \right[{\text{JUN}}\,*\,\left( {0.00 3 3 3 9 8 1 8} \right)] \\ & \, + \,\left[ {{\text{JAG1}}\,*\,\left( {0.00 2 7 30 9 1} \right)} \right]\, + \,\left[ {{\text{SPP1}}\,*\,\left( {0.000 3 8 4 1 1 3} \right)} \right]\, + \,[{\text{FGFR4}}\,*\,\left( {0.0 5 8 40 9 5 3 8} \right) \\ & \, + \,\left[ {{\text{AGTR2}}\,*\,\left( {0.0 3 3 9 9 5 6 1 4} \right)} \right]\, + \,[{\text{TNFRSF18}}\,*\,\left( { - \,0.00 8 80 7 40 1} \right)]. \\ \end{aligned}$$

According to this immune-related biomarker, the clinical outcome of high-risk and low-risk groups could be well distinguished in training and validation sets (Fig. 3c, d). The AUC value of ROC curve was 0.692, 0.702, 0.656 and 0.655, 0.551, 0.713 in training and validation set, respectively, which indicated that the prognostic features based on IRGs have moderate potential in survival monitoring (Fig. 3e, f). Univariate and multivariate Cox regression analysis showed that the prognostic indexes were independent predictors after adjusting for other parameters, for example, gender, tumor stage, age, metastasis, and lymph node (Table 1).

**Table 1:** Univariate and Multivariate regression analysis of LUSC

Variables	Univariate analysis				Multivariate analysis			
	HR	HR0.95L	HR0.95H	P value	HR	HR0.95L	HR0.95H	P value
Age	1.020950985	0.999614487	1.042742905	0.054331873	1.017961038	0.995298322	1.04113978	0.121213533
Gender	1.379009269	0.91009885	2.089516499	0.129601938	1.49490472	0.982161625	2.275328279	0.060657928
Stage	1.312726712	1.077250626	1.599675488	0.006982166	1.024047188	0.638679737	1.641938177	0.921416616
T stage	1.323372248	1.066256833	1.642488051	0.011021532	1.194741067	0.849191412	1.680900438	0.307024365
M stage	2.232878181	0.706946144	7.052510314	0.171013949	2.18843533	0.528787421	9.057040707	0.279818155
N stage	1.216897792	0.966420088	1.532294553	0.095022688	1.311029236	0.854094408	2.012421156	0.215484682
RiskScore	2.8091709	2.079248648	3.795333065	1.72E−11	2.819329395	2.082596762	3.816686161	1.98E−11

### Characteristics of survival-related IRGs

**Fig. 4:**
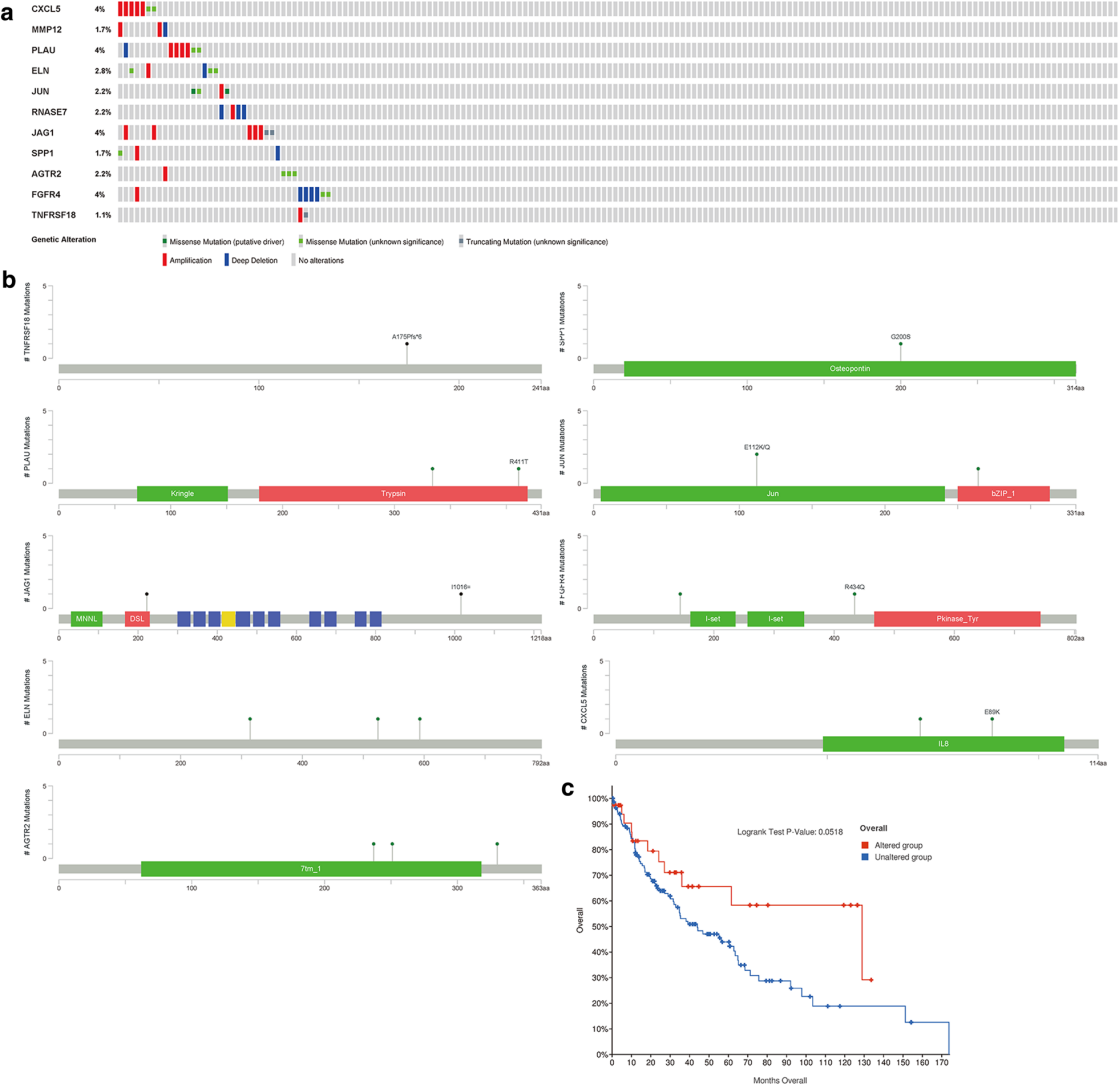
Mutations of risk genes. **a** The diagram reflected the mutation type and frequency of risk genes. **b** Gene mutation site. **c** Survival curve of LUSC patients with risk gene mutation and patients without these mutations

Identified survival-related IRGs have outstanding biomarker capacity and could be used to monitor prognosis. Most of these core IRGs were upregulated in LUSC samples (Table 2), and most of these genes were risk factors. While TNFRSF18 was defined as positive effectors. In the genetic alterations of these IRGs, deep deletion and amplification were the most common forms (Fig. 4a). CXCL5, PLAU, and FGFR4 was the gene that had the most genetic alternations. SPP1, PLAU, JUN, JAG1, CXCL5, and AGTR2 existed mutations in the protein functional domain (Fig. 4b), and these IRGs mutations may affect the prognosis of LUSC patients (Fig. 4c).

**Table 2:** General characteristics of LUSC immune-related genes

Gene symbol	HR	P value	logFC	FDR
CXCL5	1.007754	0.014338	− 1.70462	9.92E−18
MMP12	1.003131	0.010559	7.548206	1.34E−27
PLAU	1.002777	0.00243	3.249016	4.05E−23
ELN	1.008716	0.108874	− 1.61414	7.71E−19
JUN	1.003345	0.06374	− 1.06491	1.32E−12
RNASE7	1.010204	0.036693	2.835585	1.26E−17
JAG1	1.002735	0.044368	2.5708	6.35E−22
SPP1	1.000384	0.001399	5.468771	2.82E−23
AGTR2	1.03458	0.035991	− 2.5684	3.92E−24
FGFR4	1.060149	0.013799	− 2.5443	4.42E−28
TNFRSF18	0.991231	0.150625	3.205853	1.07E−24

### Clinic correlation and nomogram of immune-related genes

**Fig. 5:**
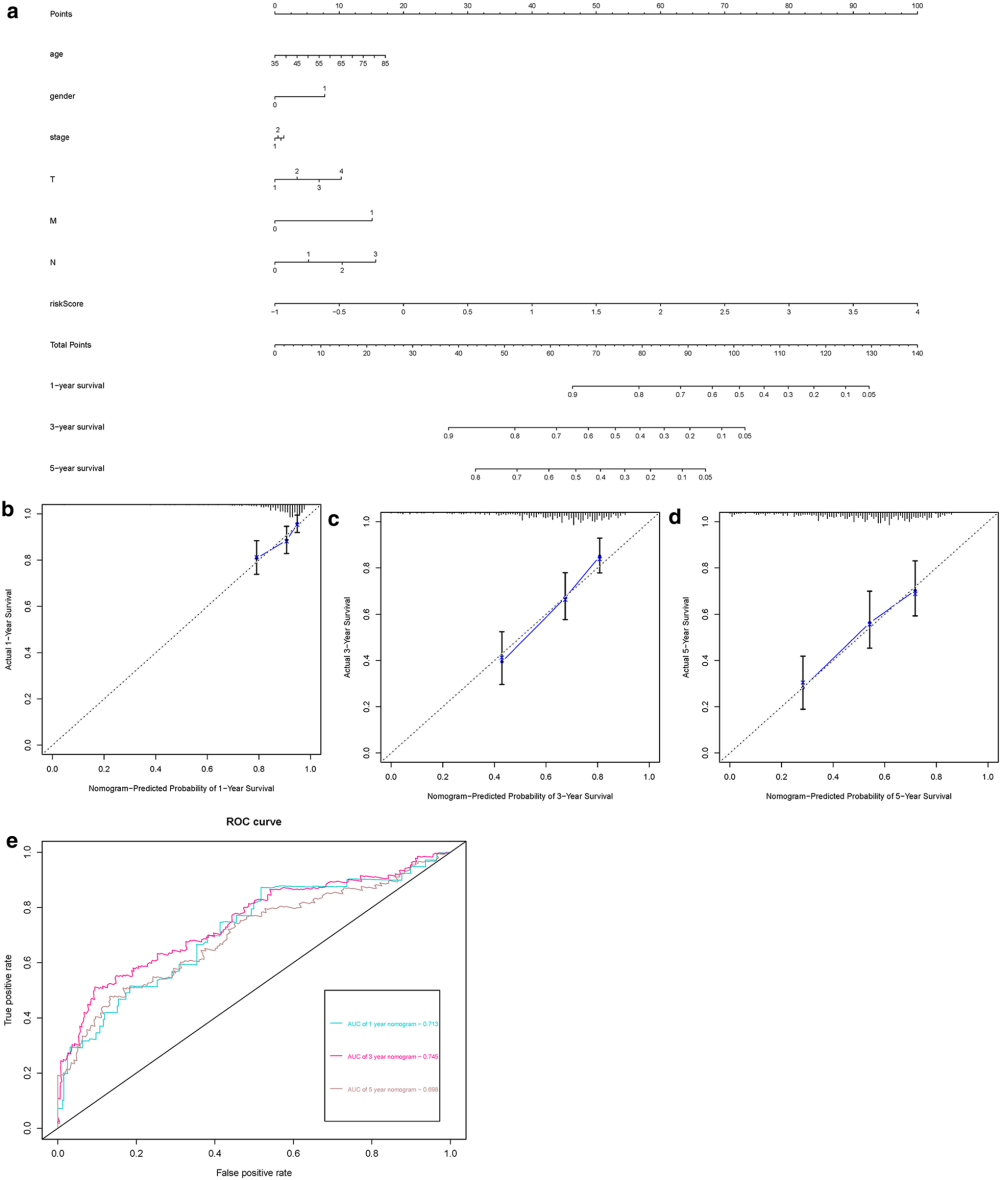
IRGs combined with other clinical factors to predict the prognosis of patients with LUSC. **a** Nomogram. **b**, **c** and **d** The calibration curve was drawn to verify the accuracy of the prediction model for predicting 1-,3-, 5-year survival rates. **e** ROC curve verifies the accuracy of the combined model in predicting the 1-, 3-, 5-year survival rates of LUSC patients

The ggpubr package was applied to explore the connection of IRGs and clinical factors (Table 3). Except for CXCL5, JAG1, and SPP1, the rest of the IRGs were related to clinical factors. At the same time, we utilized IRGs together with clinical factors to draw a nomogram (Fig. 5a) and the calibration curve was drawn to verify the accuracy of the prediction model (Fig. 5b–d). The predicted value fits well with the real value, suggesting that our model might be applied to prophesy the prognosis of LUSC patients. ROC was performed to measure the clinical effectiveness of the nomogram. For the 1-, 3-, and 5-year OS probability, the ROC curve showed that the combination of IRGs and other clinical factors were better than the model built only by IRGs (Fig. 5e).

**Table 3:** Relationships between the expressions of the immune-related genes and the clinicopathological factors in LUSC

Gene symbol	Age (≧65/< 65)		Gender (male/female)		Stage (I&II/III&IV)		T stage (T1–T2/T3–T4)		M stage (M0/M1)		N stage (N0/N1-3)	
	t	P	t	P	t	P	t	P	t	P	t	P
CXCL5	0.391	0.696	− 1.302	0.194	− 0.972	0.334	− 1.108	0.271	0.58	0.583	− 0.7	0.485
MMP12	− 2.276	*0.023*	1.365	0.174	0.518	0.605	2.307	*0.022*	0.25	0.812	− 0.927	0.355
PLAU	− 2.427	*0.016*	0.101	0.92	0.547	0.585	− 2.346	*0.021*	0.032	0.975	2.492	*0.013*
ELN	− 1.893	0.059	2.107	*0.037*	− 0.21	0.834	− 0.143	0.886	2.591	*0.031*	0.397	0.691
JUN	− 2.521	*0.012*	1.592	0.113	− 0.353	0.725	− 1.425	0.158	− 0.619	0.559	1.089	0.277
RNASE7	− 0.113	0.91	0.372	0.71	2.42	*0.016*	− 0.596	0.553	3.296	*0.007*	0.094	0.926
JAG1	− 0.625	0.532	− 1.557	0.121	− 0.903	0.369	− 0.85	0.398	− 1.501	0.189	− 0.837	0.404
SPP1	0.261	0.794	− 1.242	0.216	− 0.855	0.395	0.131	0.896	0.133	0.899	0.936	0.35
AGTR2	− 0.707	0.48	0.086	0.932	1.931	0.054	− 0.009	0.993	2.317	*0.036*	*3.376*	*8.28E−04*
FGFR4	1.423	0.156	− 2.509	*0.013*	− 0.89	0.376	0.632	0.528	9.289	*7.33E−14*	− 1.725	*0.086*
TNFRSF18	1.752	*0.081*	− 0.933	0.352	− 0.29	0.773	− 0.24	0.811	0.077	0.941	− 0.81	0.419
riskScore	− 2.79	*0.006*	0.267	0.79	− 0.596	0.553	− 1.255	0.213	1.597	0.159	1.188	0.236

### Validation of the immune-related genes

**Fig. 6:**
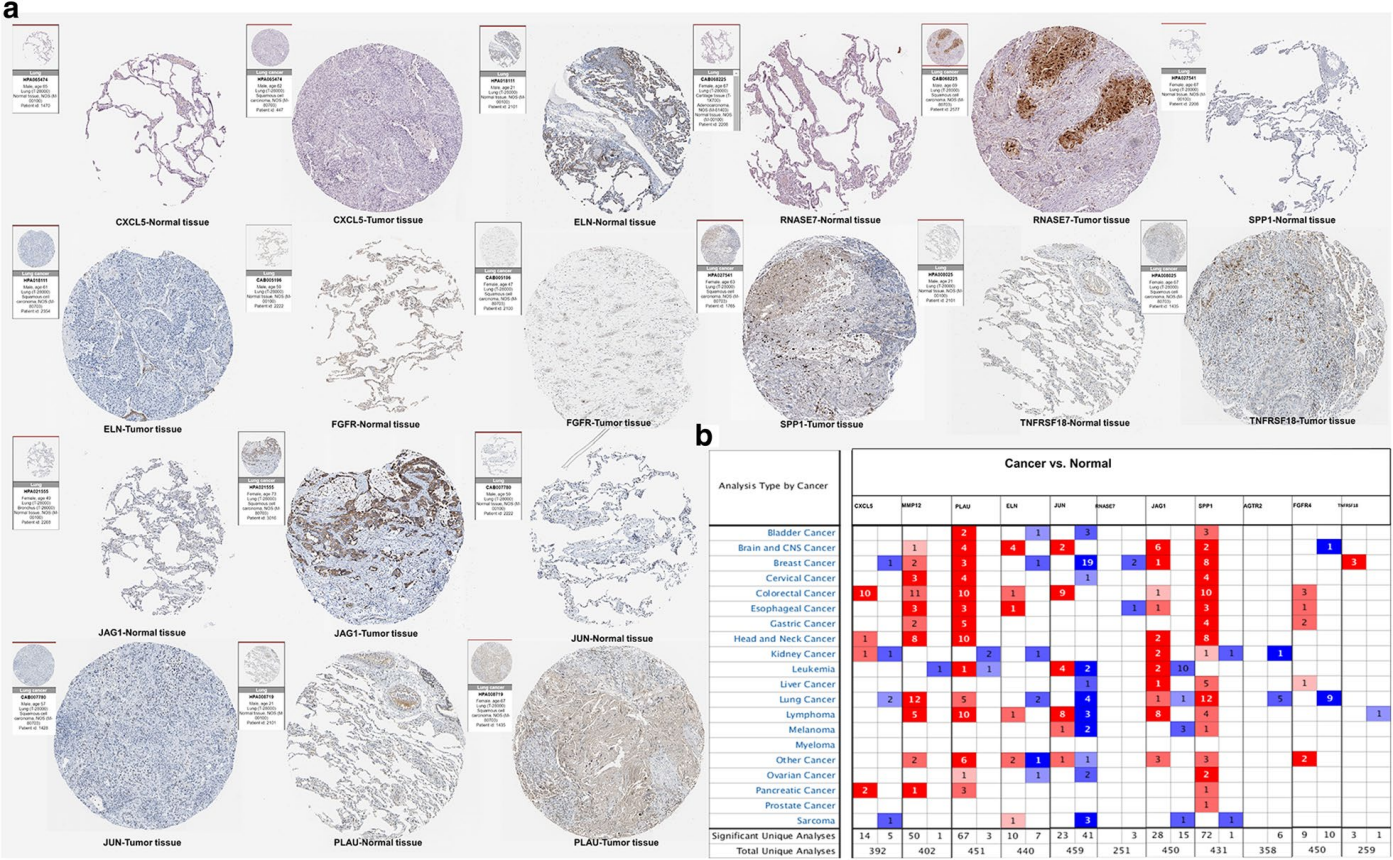
Validation of the IRGs. **a** Immunohistochemistry of the IRGs based on the Human Protein Atlas. **b** The mRNA expression patterns of IRGs in the pan- cancers. The differences in mRNA expression between tumors and normal tissues were analyzed by the Oncomine database

Based on the HPA database, the function of IRGs was verified at the protein levels by immunohistochemistry (Fig. 6a). The results were accordant with our preceding research. CXCL5, ELN, JUN, and FGFR4 were highly expressed in normal tissues, while PLAU, RNASE7, JAG1, SPP1, and TNFRSF18 were highly expressed in tumor tissues. Oncomine analysis of tumor and normal tissues (Fig. 6b) showed that the expression patterns of some IRGs in LUSC were different from those in other tumors.

### Immunocyte infiltration in the tumor microenvironment

**Fig. 7:**
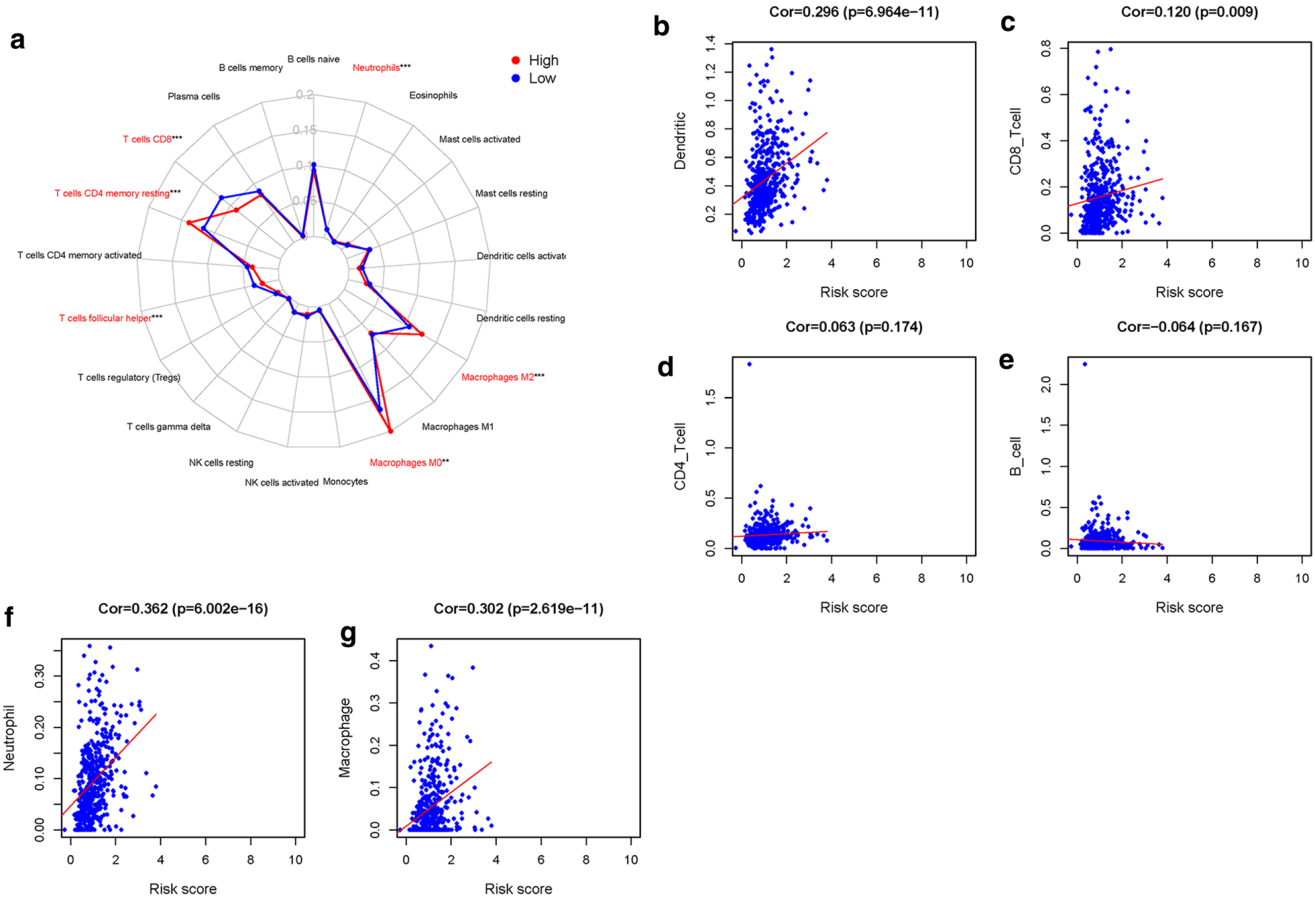
Immune cell infiltration. **a** Radar plot of low-risk groups and high-risk groups with LUSC, blue stand for low-risk groups and red represent the high-risk groups. Analysis of the correlation between risk score and immune cells. **b** Dendritic cells. **c** CD8 + T cells. **d** CD4 + T cells. **e** B cells. **f** Neutrophils. **g** Macrophages

To understand whether the immune genome exactly mirrored the condition of the LUSC immune microenvironment, we analyzed the connection between IRGs and immune cell infiltration by CIBERSORT algorithm and TIMER database. The proportion of 22 kinds of immune cells in LUSC was calculated through CIBERSORT algorithm. We found that resting memory CD4 + T cell, M0 macrophage, M2 macrophage, and neutrophil infiltration levels were higher in high-risk group. While T cell follicular helper, and CD8 + T cells were higher in low-risk group (Fig. 7a). The TIMER database was also applied to investigate the connection of the IRGs and immune cell infiltration. CD4 + T cells, CD8 + T cells, dendritic cells, neutrophils, and macrophages were positively related to IRGs (Fig. 7b–g).

### Immune status analysis for high-risk and low-risk groups

**Fig. 8:**
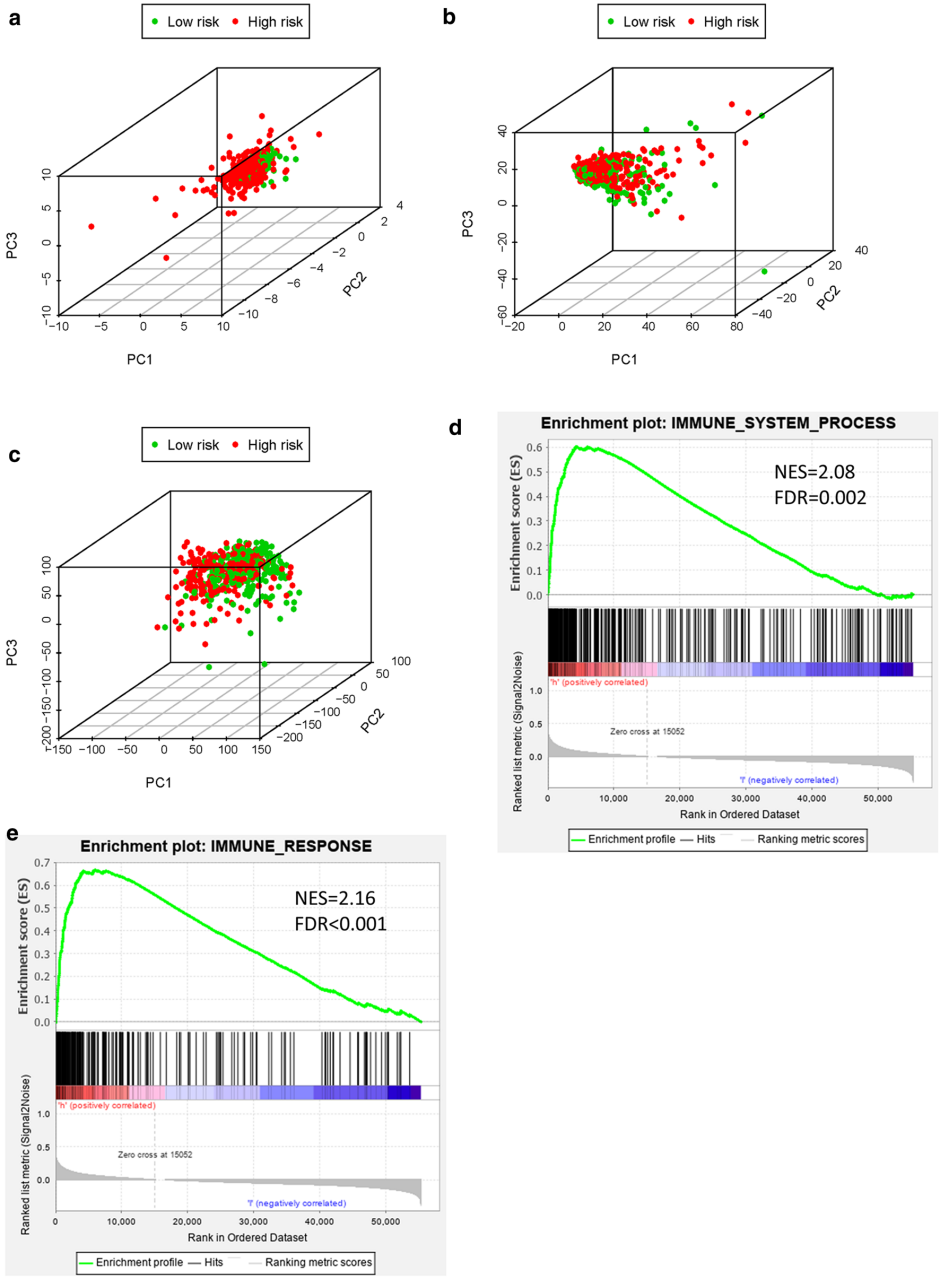
The high- and low-risk groups showed different distribution patterns and gene-set enrichment analysis. **a** PCA of the high- and low-risk groups based on the 11 risk genes. **b** PCA of the high- and low-risk groups based on the whole immune-genome set. **c** PCA of the high- and low-risk groups based on the whole genome set. **d** Immune system process and **e** immune response enrichment analysis by GSEA

To study whether the LUSC patients could be distinguished properly based on our prognosis model, PCA analysis was utilized to explore the distinct distribution modes between the high-risk groups and low-risk groups. According to risk genes, the high-risk and low-risk groups tend to be divided into two aspects (Fig. 8a). Based on the whole-genome sets and whole-IRGs, high-risk and low-risk groups did not show significant separation in immune status (Fig. 8b, c), while the model based on our risk genes could well distinguish the difference of immune status between high- and low-risk groups. The GSEA further validated the functional annotations and found that the high-risk groups had more immune responses than the low-risk groups (immune system process, NES = 2.08, FDR = 0.002; immune response, NES = 2.16, FDR < 0.001; Fig. 8d, e). These results were consistent with the results of immune cell infiltration, indicating that the high-risk scores were correlated with the enhanced immunophenotype.

### Correlation analysis of immune checkpoints and risk score

**Fig. 9:**
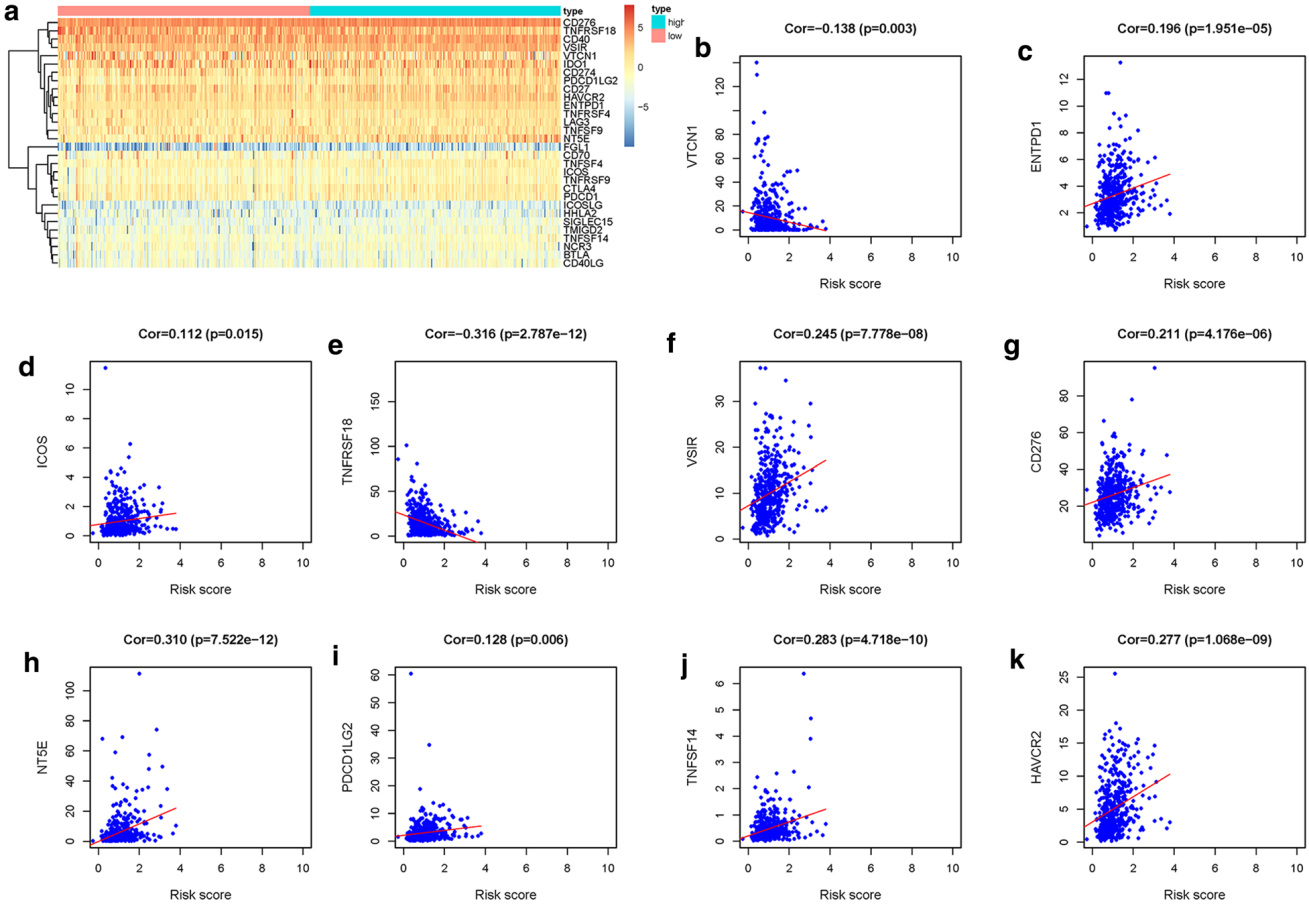
Immune checkpoint. **a** Heatmap of 30 immune checkpoints between low- and high-risk groups. Analysis of the correlation between risk score and immune checkpoints. **b** VTCN1. **c** ENTPD1. **d** ICOS. **e** TNFRSF18. **f** VSIR. **g** CD276. **h** NT5E. **i** PDCD1LG2. **j** TNFSF14. **k** HAVCR2

To investigate the relationship between risk score and immune checkpoint, we extracted the expression of 30 immune checkpoint (Fig. 9a), including B7-CD28 family (CD274, CD276, CTLA4, HHLA2, ICOS, ICOSLG, PDCD1, PDCD1LG2, TMIGD2, VTCN1), TNF superfamily (BTLA, CD27, CD40LG, CD40, CD70, TNFRSF18, TNFRSF4, TNFRSF9, TNFSF14, TNFSF4, TNFSF9), and other immune checkpoint (HAVCR2, IDO1, LAG3, FGL1, ENTPD1, NT5E, SIGLEC15, VSIR, NCR3). We then calculated the correlation between risk score and the expression of immune checkpoint. The results illustrated that ICOS, NT5E, PDCD1LG2, ENTPD1, VSIR, CD276, TNFSF14, HAVCR2 were positively correlated with risk score, while TNFRSF18 and VTCN1 were negatively correlated with risk score (Fig. 9b–k).

## Discussion

The importance of IRGs in cancer deterioration and immunotherapy has been accepted, but overall genome-wide analysis is still to be investigated to explore the molecular mechanism and clinical significance. Our researches revealed the effects of IRGs on LUSC clinical significance and elucidated the molecular characteristics. these IRGs might be valuable clinical indicators. Personalized immune-related prognostic characteristics on the basis of selective, differentially expressed IRGs were raised to evaluate potential clinical outcomes and measure immune cell infiltration.

To establish a suitable and simple scheme to observe the immune status of LUSC patients and imply clinical outcomes, we built an IRGs-based prognostic index. With the consequences of multivariate regression analysis, the prognostic indexes based on 11 IRGs (CXCL5, MMP12, PLAU, ELN, JUN, RNASE7, JAG1, SPP1, AGTR2, FGFR4, and TNFRSF18) were established. Patients with high-risk values have a bad prognosis, whose survival time was shortened with increased risk values. Moreover, univariate COX and multivariate COX regression analysis illustrated that the prognostic signature based on these IRGs might be applied as independent prognostic factors. We also constructed a nomograph composed of IRGs and other clinical factors to predict the OS. Our studies suggested that IRGs could be used as prognostic markers and indexes of immune status.

The TF-IRG regulatory network based on CHIP-SEQ and co-expression will assist to guide and inform the analysis of future mechanisms. The mechanism and function of RNASE7, and ELN had not been reported in lung cancer. The other 9 IRGs, including MMP12, PLAU, JUN, TNFRSF18, JAG1, FGFR4, AGTR2, CXCL5, and SPP1. CXCL5, FGFR4, and PLAU were reported to be a potential prognostic factor in LUSC [35]. Ella et al. suggest that overexpression of MMP12 promotes invasion and migration of lung cancer cells [36]. Chang et al. found that the ectopic expression of JAG1 in lung cancer cells enhances cell migration, invasion and metastasis in vivo and in vitro [37]. It was reported that SPP1 could not only be used as a prognostic biomarker of lung cancer but also play a role in mediating macrophage polarization and immune escape [38, 39]. In small cell lung cancer, TNFRSF18 has been found to bind to its receptor and induce apoptosis [40]. The complex of dTAT- AGTR2-Ca2 + could inhibit the growth of Lewis lung carcinoma in mice [41]. Yang et al. discovered that JUN played a role in the inhibition of growth and apoptosis of NSCLC by PS-341 [42]. However, these studies offered a finite message on the mechanism of 11 IRGs in the survival of LUSC patients.

To establish a suitable and simple scheme to observe the immune status of LUSC patients and imply clinical outcomes, we established an immune-based prognostic index. Shi et al. investigated DNA methylation profiling and put forward potential diagnostic biomarkers for LUSC [43]. Chen et al. investigated the roles of IRGs in deterioration of lung cancer and indicated the distinct between LUAD and LUSC from the perspective of the immune response [44]. Several researchers have put forward a prognostic marker for survival prediction in patients with LUSC [45–47]. Our study suggested that IRGPI could be used as a prognostic marker. In addition, it could also be utilized as an index of immune status.

On the basis of 11 IRGs in LUSC, this prognostic indicator showed satisfactory clinical feasibility. The tool could adjust the treatment plan relatively quickly according to the level of immune cell infiltration reflected by IRGPI. The results of TIMER database showed that these genes were positively related to the infiltration of CD4 + T cells, CD8 + T cells, dendritic cells, neutrophils, and macrophages. However, the results of CIBERSORT showed that CD8 + T cells infiltrated more in the low-risk group, which was different from the TIMER database, resulting from the difference between the 2 algorithms. TIMER quantified 6 kinds of immune cells, but it was different from CIBERSORT (CIBERSORT analysis: the total proportion of 22 kinds of immune cells added to 100%). TIMER did not standardize the predicted value to 1. Therefore, the results could not be interpreted as cell fraction or compared in different data sets. The imbalance of immune cell composition was associated with the survival rate and bad prognosis of cancer patients [48]. Our findings expanded and confirmed the discovery that the infiltration level of the immune cells was crucial for the progression of LUSC. The result indicated that these IRGs had the capacity to be a predictor of increased infiltration of immune cells, which was consistent with previous reports. Nevertheless, the function of the infiltration immune cell of LUSC was still unknown. Our initial observations offered an opinion for investigating this issue, and further study was required in the future.

Interestingly, we found that the risk score was not only associated with immune cell infiltration, but also correlated with the expression of immune checkpoint genes. CD276, PDCD1LG2 (PD-L2) were a member of the B7 transmembrane glycoprotein family, and their expression were associated with poor prognosis and tumor immune escape of NSCLC [49–51]. VTCN1 also belonged to the B7 family, but its expression was not related to B cell and T cell infiltration in lung cancer [52]. It was reported that TNFSF14, a member of Tumor necrosis factor superfamily, played an important role in Osteolytic Bone Metastases of NSCLC patients [53]. HAVCR2, also known as TIM-3, was mainly distributed in NK cells and macrophages in NSCLC, which could suppress anti-tumor immunity [54]. Franz et al. found that VSIR was related to the increase of lymphocyte infiltration in tumor microenvironment, specific gene mutation and prognosis of NSCLC patients [55]. The function of NT5E was hydrolyzed extracellular nucleotides, overexpression of which gene could inhibit anti-tumor immune response and contribute to proliferation, angiogenesis, and metastasis [56]. With the increase of risk score, the gene expression in most immune checkpoints increased, which was consistent with the poor prognosis of patients in the high-risk group. Meanwhile, the treatment of these immune checkpoints may improve the prognosis of LUSC patients.

However, the current study had some shortcomings, which ought to be taken into consideration when explaining our results. First of all, transcriptome analysis could only reflect certain aspects of the immune state, but not global changes. Secondly, the verification with another independent queue was lacked. At the last, our results also required validation of in vivo and in vitro experiments.

## Conclusion

On the basis of gene sets downloaded from the TCGA and GEO database, we utilized LASSO regression analysis and univariate cox regression analysis in R to screen IRGs associated with the prognosis of LUSC patients. A prediction model was constructed based on 11 IRGs (CXCL5, MMP12, PLAU, ELN, JUN, RNASE7, JAG1, SPP1, AGTR2, FGFR4, and TNFRSF18). This model also well predicted immune cell infiltration in LUSC. Our study provided a potential model and biomarkers for further immune-related work and personalized medicine for the treatment of LUSC.

## Data Availability

All data generated or analysed in this study are included in this published article.

## Abbreviations

TCGA: The cancer genome atlas
IRGS: Immune-related genes
OS: Overall survival
GSEA: Gene set enrichment analysis
LUSC: Lung squamous cell carcinoma
NSCLC: Non-small cell lung cancer
PD-1: Cell death protein 1
PD-L1: PD-1 ligand 1
CTLA-4: Cytotoxic T lymphocyte antigen-4
ImmPort: Immunology database and analysis portal
GO: Gene ontology
KEGG: Kyoto encyclopedia of genes and genomes
IRGPI: Immune-related gene-based prognostic index

## References

[1] Muller DC, Larose TL, Hodge A, Guida F, Langhammer A, Grankvist K, Meyer K, Cai QY, Arslan AA, Zeleniuch-Jacquotte A (2019). Circulating high sensitivity C reactive protein concentrations and risk of lung cancer: nested case-control study within lung cancer cohort consortium. Bmj-Brit Med J.

[2] Blandin S, Crosbie PA, Balata H, Chudziak J, Hussell T, Dive C (2017). Progress and prospects of early detection in lung cancer. Open Biol.

[3] Travis WD, Brambilla E, Nicholson AG, Yatabe Y, Austin JHM, Beasley MB, Chirieac LR, Dacic S, Duhig E, Flieder DB (2015). The 2015 world health organization classification of lung tumors impact of genetic, clinical and radiologic advances since the 2004 classification. J Thorac Oncol.

[4] Hirsch FR, Spreafico A, Novello S, Wood MD, Simms L, Papotti M (2008). The prognostic and predictive role of histology in advanced non-small cell lung cancer: a literature review. J Thorac Oncol.

[5] Rosado-de-Christenson ML, Templeton PA, Moran CA (1994). Bronchogenic carcinoma: radiologic-pathologic correlation. Radiographics.

[6] Nichols L, Saunders R, Knollmann FD (2012). Causes of death of patients with lung cancer. Arch Pathol Lab Med.

[7] Siegel RL, Miller KD, Jemal A (2019). Cancer statistics, 2019. CA Cancer J Clin.

[8] Kobold S, Pantelyushin S, Rataj F, Vom Berg J (2018). Rationale for combining bispecific T cell activating antibodies with checkpoint blockade for cancer therapy. Front Oncol.

[9] Popovic A, Jaffee EM, Zaidi N (2018). Emerging strategies for combination checkpoint modulators in cancer immunotherapy. J Clin Invest.

[10] Li S, Yang F, Ren X (2015). Immunotherapy for hepatocellular carcinoma. Drug Discov Ther.

[11] Carter BW, Halpenny DF, Ginsberg MS, Papadimitrakopoulou VA, de Groot PM (2017). Immunotherapy in non-small cell lung cancer treatment: current status and the role of imaging. J Thorac Imag.

[12] Fridman WH, Pages F, Sautes-Fridman C, Galon J (2012). The immune contexture in human tumours: impact on clinical outcome. Nat Rev Cancer.

[13] Oja AE, Piet B, van der Zwan D, Blaauwgeers H, Mensink M, de Kivit S, Borst J, Nolte MA, van Lier RAW, Stark R (2018). Functional heterogeneity of CD4(+) tumor-infiltrating lymphocytes with a resident memory phenotype in NSCLC. Front Immunol.

[14] Bruno TC, Ebner PJ, Moore BL, Squalls OG, Waugh KA, Eruslanov EB, Singhal S, Mitchell JD, Franklin WA, Merrick DT (2017). Antigen-presenting intratumoral B cells affect CD4(+) TIL phenotypes in non-small cell lung cancer patients. Cancer Immunol Res.

[15] Hiraoka K, Miyamoto M, Cho Y, Suzuoki M, Oshikiri T, Nakakubo Y, Itoh T, Ohbuchi T, Kondo S, Katoh H (2006). Concurrent infiltration by CD8(+) T cells and CD4(+) T cells is a favourable prognostic factor in non-small-cell lung carcinoma. Brit J Cancer.

[16] Bos R, Sherman LA (2010). CD4(+) T-cell help in the tumor milieu is required for recruitment and cytolytic function of CD8(+) T lymphocytes. Cancer Res.

[17] Nakanishi Y, Lu B, Gerard C, Iwasaki A (2009). CD8(+) T lymphocyte mobilization to virus-infected tissue requires CD4(+) T-cell help. Nature.

[18] Rakhra K, Bachireddy P, Zabuawala T, Zeiser R, Xu LW, Kopelman A, Fan AC, Yang QW, Braunstein L, Crosby E (2010). CD4(+) T cells contribute to the remodeling of the microenvironment required for sustained tumor regression upon oncogene inactivation. Cancer Cell.

[19] Yuan C, Xiang LY, Bai R, Cao K, Gao YP, Jiang XP, Zhang NN, Gong Y, Xie CH (2019). MiR-195 restrains lung adenocarcinoma by regulating CD4 + T cell activation via the CCDC88C/Wnt signaling pathway: a study based on the Cancer Genome Atlas (TCGA), Gene Expression Omnibus (GEO) and bioinformatic analysis. Ann Transl Med.

[20] Schadendorf D, Hodi FS, Robert C, Weber JS, Margolin K, Hamid O, Patt D, Chen TT, Berman DM, Wolchok JD (2015). Pooled analysis of long-term survival data from phase II and phase III trials of ipilimumab in unresectable or metastatic melanoma. J Clin Oncol.

[21] Liu X, Cho WC (2017). Precision medicine in immune checkpoint blockade therapy for non-small cell lung cancer. Clin Transl Med.

[22] Remon J, Besse B (2017). Immune checkpoint inhibitors in first-line therapy of advanced non-small cell lung cancer. Curr Opin Oncol.

[23] Schumacher TN, Schreiber RD (2015). Neoantigens in cancer immunotherapy. Science.

[24] Liu XY, Wu SC, Yang YH, Zhao M, Zhu GY, Hou ZH (2017). The prognostic landscape of tumor-infiltrating immune cell and immunomodulators in lung cancer. Biomed Pharmacother.

[25] Li B, Cui Y, Diehn M, Li R (2017). Development and validation of an individualized immune prognostic signature in early-stage nonsquamous non-small cell lung cancer. JAMA Oncol.

[26] The Cancer Genome Atlas [https://cancergenome.nih.gov/] Accessed 24 Sep 2019.

[27] Bhattacharya S, Andorf S, Gomes L, Dunn P, Schaefer H, Pontius J, Berger P, Desborough V, Smith T, Campbell J (2014). ImmPort: disseminating data to the public for the future of immunology. Immunol Res.

[28] Wang L, Shi J, Huang Y, Liu S, Zhang J, Ding H, Yang J, Chen Z (2019). A six-gene prognostic model predicts overall survival in bladder cancer patients. Cancer Cell Int.

[29] Heagerty PJ, Lumley T, Pepe MS (2000). Time-dependent ROC curves for censored survival data and a diagnostic marker. Biometrics.

[30] Gao JJ, Aksoy BA, Dogrusoz U, Dresdner G, Gross B, Sumer SO, Sun YC, Jacobsen A, Sinha R, Larsson E (2013). Integrative analysis of complex cancer genomics and clinical profiles using the cBioPortal. Sci Signal.

[31] Cerami E, Gao J, Dogrusoz U, Gross BE, Sumer SO, Aksoy BA, Jacobsen A, Byrne CJ, Heuer ML, Larsson E (2012). The cBio cancer genomics portal: an open platform for exploring multidimensional cancer genomics data. Cancer Discov.

[32] Cbioportal [http://www.cbioportal. org/] Accessed 1 October 2019.

[33] Mei S, Meyer CA, Zheng R, Qin Q, Wu Q, Jiang P, Li B, Shi X, Wang B, Fan J (2017). Cistrome cancer: a web resource for integrative gene regulation modeling in cancer. Cancer Res.

[34] gene set enrichment analysis [http://www.broadinstitute.org/gsea/index] Accessed 3 Oct 2019.

[35] Kowalczuk O, Burzykowski T, Niklinska WE, Kozlowski M, Chyczewski L, Niklinski J (2014). CXCL5 as a potential novel prognostic factor in early stage non-small cell lung cancer: results of a study of expression levels of 23 genes. Tumor Biol.

[36] Ella E, Harel Y, Abraham M, Wald H, Benny O, Karsch-Bluman A, Vincent D, Laurent D, Amir G, Izhar U (2018). Matrix metalloproteinase 12 promotes tumor propagation in the lung. J Thorac Cardiovasc Surg.

[37] Chang WH, Ho BC, Hsiao YJ, Chen JS, Yeh CH, Chen HY, Chang GC, Su KY, Yu SL (2016). JAG1 Is associated with poor survival through inducing metastasis in lung cancer. Plos One.

[38] Li S, Yang R, Sun X, Miao S, Lu T, Wang Y, Wo Y, Jiao W (2018). Identification of SPP1 as a promising biomarker to predict clinical outcome of lung adenocarcinoma individuals. Gene.

[39] Zhang Y, Du W, Chen Z, Xiang C (2017). Upregulation of PD-L1 by SPP1 mediates macrophage polarization and facilitates immune escape in lung adenocarcinoma. Exp Cell Res.

[40] Kopru CZ, Cagnan I, Akar I, Esendagli G, Korkusuz P, Gunel-Ozcan A (2018). Dual effect of glucocorticoid-induced tumor necrosis factor-related receptor ligand carrying mesenchymal stromal cells on small cell lung cancer: a preliminary in vitro study. Cytotherapy.

[41] Ishiguro S, Alhakamy NA, Uppalapati D, Delzeit J, Berkland CJ, Tamura M (2017). Combined local pulmonary and systemic delivery of AT2R gene by modified TAT peptide nanoparticles attenuates both murine and human lung carcinoma Xenografts in Mice. J Pharm Sci.

[42] Yang Y, Ikezoe T, Saito T, Kobayashi M, Koeffler HP, Taguchi H (2004). Proteasome inhibitor PS-341 induces growth arrest and apoptosis of non-small cell lung cancer cells via the JNK/c-Jun/AP-1 signaling. Cancer Sci.

[43] Shi YX, Wang Y, Li X, Zhang W, Zhou HH, Yin JY, Liu ZQ (2017). Genome-wide DNA methylation profiling reveals novel epigenetic signatures in squamous cell lung cancer. BMC Genomics.

[44] Chen MZ, Liu XY, Du J, Wang XJ, Xia LX (2017). Differentiated regulation of immune-response related genes between LUAD and LUSC subtypes of lung cancers. Oncotarget.

[45] Zhang J, Bing Z, Yan P, Tian J, Shi X, Wang Y, Yang K (2019). Identification of 17 mRNAs and a miRNA as an integrated prognostic signature for lung squamous cell carcinoma. J Gene Med.

[46] Mavridis K, Gueugnon F, Petit-Courty A, Courty Y, Barascu A, Guyetant S, Scorilas A (2015). The oncomiR miR-197 is a novel prognostic indicator for non-small cell lung cancer patients. Brit J Cancer.

[47] Ning PB, Wu ZX, Hu AX, Li XP, He J, Gong XC, Xia YQ, Shang YK, Bian HJ (2018). Integrated genomic analyses of lung squamous cell carcinoma for identification of a possible competitive endogenous RNA network by means of TCGA datasets. Peerj.

[48] Bense RD, Sotiriou C, Piccart-Gebhart MJ, Haanen J, van Vugt M, de Vries EGE, Schroder CP, Fehrmann RSN (2017). Relevance of tumor-infiltrating immune cell composition and functionality for disease outcome in breast cancer. J Natl Cancer Inst.

[49] Zhang C, Hao X (2019). Prognostic significance of CD276 in non-small cell lung cancer. Open Med.

[50] Yonesaka K, Haratani K, Takamura S, Sakai H, Kato R, Takegawa N, Takahama T, Tanaka K, Hayashi H, Takeda M (2018). B7-H3 negatively modulates CTL-mediated cancer immunity. Clin Cancer Res.

[51] Tanegashima T, Togashi Y, Azuma K, Kawahara A, Ideguchi K, Sugiyama D, Kinoshita F, Akiba J, Kashiwagi E, Takeuchi A (2019). Immune suppression by PD-L2 against spontaneous and treatment-related antitumor immunity. Clin Cancer Res.

[52] Schalper KA, Carvajal-Hausdorf D, McLaughlin J, Altan M, Velcheti V, Gaule P, Sanmamed MF, Chen L, Herbst RS, Rimm DL (2017). Differential expression and significance of PD-L1, IDO-1, and B7-H4 in human lung cancer. Clin Cancer Res.

[53] Brunetti G, Belisario DC, Bortolotti S, Storlino G, Colaianni G, Faienza MF, Sanesi L, Alliod V, Buffoni L, Centini E (2020). LIGHT/TNFSF14 promotes osteolytic bone metastases in non-small cell lung cancer patients. J Bone Miner Res.

[54] Datar I, Sanmamed MF, Wang J, Henick BS, Choi J, Badri T, Dong W, Mani N, Toki M, Mejias LD (2019). Expression analysis and significance of PD-1, LAG-3, and TIM-3 in human non-small cell lung cancer using spatially resolved and multiparametric single-cell analysis. Clin Cancer Res.

[55] Villarroel-Espindola F, Yu X, Datar I, Mani N, Sanmamed M, Velcheti V, Syrigos K, Toki M, Zhao H, Chen L (2018). Spatially resolved and quantitative analysis of VISTA/PD-1H as a novel immunotherapy target in human non-small cell lung cancer. Clin Cancer Res.

[56] Ghalamfarsa G, Kazemi MH, Raoofi Mohseni S, Masjedi A, Hojjat-Farsangi M, Azizi G, Yousefi M, Jadidi-Niaragh F (2019). CD73 as a potential opportunity for cancer immunotherapy. Expert Opin Ther Targets.

